# Fluorescent non transgenic schistosoma to decipher host-parasite phenotype compatibility

**DOI:** 10.3389/fimmu.2023.1293009

**Published:** 2023-11-27

**Authors:** David Duval, Pierre Poteaux, Benjamin Gourbal, Anne Rognon, Ronaldo De Carvalho Augusto

**Affiliations:** IHPE, Université de Perpignan Via Domitia, CNRS, Ifremer, Université de Montpellier, Perpignan, France

**Keywords:** biomphalaria, host/parasite interaction, fluorescent vital probe, Schistosoma, vibratome section, 3D histochemistry

## Abstract

Schistosomiasis is considered as a significant public health problem, imposing a deeper understanding of the intricate interplay between parasites and their hosts. Unfortunately, current invasive methodologies employed to study the compatibility and the parasite development impose limitations on exploring diverse strains under various environmental conditions, thereby impeding progress in the field. In this study, we demonstrate the usefulness for the trematode parasite *Schistosma mansoni*, leveranging a fluorescence-imaging-based approach that employs fluorescein 5-chloromethylfluorescein diacetate (CMFDA) and 5-chloromethylfluorescein diacetate (CMAC) as organism tracker for intramolluscan studies involving the host snail *Biomphalaria glabrata.* These probes represent key tools for qualitatively assessing snail infections with unmatched accuracy and precision. By monitoring the fluorescence of parasites within the snail vector, our method exposes an unprecedented glimpse into the host-parasite compatibility landscape. The simplicity and sensitivity of our approach render it an ideal choice for evolutionary studies, as it sheds light on the intricate mechanisms governing host-parasite interactions. Fluorescent probe-based methods play a pivotal role in characterizing factors influencing parasite development and phenotype of compatibility, paving the way for innovative, effective, and sustainable solutions to enhance our understanding host-parasite immunobiological interaction and compatibility.

## Introduction

Schistosomiasis is considered as a neglected human parasitic disease that however affects more than 240 million people worldwide, predominantly in developing countries (1). The disease is caused by a flatworm parasite of the *schistosoma* genus, which has a complex life cycle that involves freshwater snails as intermediate hosts and mammals as definitive hosts. Currently, the sole chemotherapy employed in the treatment of schistosomiasis, the Praziquantel, is limited in its effectiveness as it targets exclusively adult worms and does not prevent reinfection (2). To address these challenges, a growing number of studies are seeking to expand the current approach through novel strategies, specifically by advancing our understanding of the interplay between the intermediate snail host and the parasite (3, 4). In contact with freshwater, *Schistosoma* sp. eggs release ciliated larvae called miracidia, which seek to invade a compatible snail host and developed in the first intramolluscan parasite stage called the mother sporocysts (or primary sporocysts). Each mother sporocyst can give rise to hundreds of daughters sporocysts (or secondary sporocysts), which can eventually transform into thousands of cercariae, the third intra-molluscan stage of the parasite that are dedicated to infect the definitive mammalian host by being released in the fresh water environment. Sporocysts are sac-like bodies enriched in stem cells with a phase of rapid asexual proliferation before undergoing embryogenesis to generate hundreds of new generations of either daughter sporocysts or cercariae. Indeed, from a single miracidium an virtually unlimited number of clonal sporocysts can be generated and even transplanted between snails for many generations (5–7). In the *Biomphalaria glabrata-Schistosoma mansoni* model, the concept of compatibility has been described as a complex molecular dialog between both protagonists involving multiple molecules with distinct roles (8–19). The parasite antigens *S. mansoni* polymorphic mucins (*Sm*PoMucs) and the *B. glabrata* immune receptors fibrinogen-related proteins (FREPs), two repertoires of polymorphic interacting molecules were proposed as the major components for defining the compatible/incompatible status of a specific snail/schistosome combination (18, 20, 21).

Hence sporocyst developmental trajectory is not exclusively dependent on the parasite genomic background, subsequently, it is also impacted by intrinsic factors from snail host, coinfections, or environmental stressors (22, 23–25). Individual parasites that enter the same snail can exhibit different fates; some may develop, and others may become encapsulated and killed by humoral factors. This suggests that infectivity is not a general characteristic of the parasite, but rather, it depends on the genotype of the specific host it enters. Similarly, susceptibility to infection is not a general characteristic of the host; instead, it is influenced by the genotype of the parasite it harbors. Moreover, environmental cues, such as coinfections to different parasite species, can modify the host phenotype by activating or inhibiting cellular and immune functions. Overall, the multifactorial nature of compatibility and the specificity of host-parasite interactions are crucial aspects when investigating *B.glabrata-S.mansoni* model. Understanding the mechanisms that govern the interaction between schistosomes and their intermediate snail hosts is a fundamental requirement for developing efficacious control strategies, as the exponential escalation of parasite numbers heightens the risk of disease propagation.

We theorize the use of fluorescent cell trackers as a non-invasive method in *B. glabrata-S. mansoni* compatibility studies. Fluorescent probes are valuable tools to understand cell-to-cell interaction which enable visualization and tracking *in vivo* and *in vitro* systems. These assays are instrumental in elucidating the behavior of specific pathogens within a heterogeneous milieu, providing insights into cell proliferation, viability, cytotoxicity, and motility. Fluorescent probes have been successfully used for unicellular parasites such as Trypanosoma, Leishmania, and Entamoeba sp. (26–28) and on multicellular parasites such as cestodes and nematodes to follow migration through hosts tissues, on studies of competition between different parasite species or to measure physiological stress (29–33). In these aspects, different vital stains offer the potential for labeling and monitoring cells *in vivo* and also being able to distinguish live parasites from dead ones (31). It is desirable to have tracking agents which have long-term stability, are non-toxic, and do not affect cell function. Here, we selected two different labels: CellTracker™ Green CMFDA (5-chloromethylfluorescein diacetate) and CMAC (5-chloromethylfluorescein diacetate) and performed an extensive analysis of their influence on the free-swimming parasite stage of *S. mansoni*, miracidium, as well as trackers sporocyst development into *B. glabrata* snail host.

Our results demonstrated that the fluorescent non-transgenic schistosome technique may have broad applications in the field of parasitology, as it provides a non-invasive, sensitive, and specific tool for tracking free-swimming parasites, parasite stages within snail-host. Moreover, even with the recent progress in the Crispr method, a single study managed to exhibit the feasibility of knocking along with GFP insertion in developing schistosome eggs (34). While this approach offers the advantage of visualizing fluorescence within the parasite, it demands cutting-edge molecular biology skills and comes with a substantial cost, rendering its implementation impractical in regions pivotal to the schistosomiasis eradication efforts. Our findings have the potential to provide valuable insights into the mechanisms underlying the compatibility of *Biomphalaria glabrata-Schistosoma mansoni* model.

## Materials and methods

### Ethics statement

Our laboratory holds permit #39910-2022121915564694 (APAFIS number) for experiments on animals from both the French Ministry of Agriculture and Fisheries, and the French Ministry of National Education, Research, and Technology. The housing, breeding, and animal care of the utilized animals followed the ethical requirements of our country. The researchers also possess an official certificate for animal experimentation from both French ministries (Decree # 87–848, October 19, 1987). Animal experimentation followed the guidelines of the French CNRS. The different protocols used in this study had been approved by the French veterinary agency from the DDPP Languedoc-Roussillon (Direction Départementale de Protection des Populations), Montpellier, France (authorization # 007083) and the Ethic committee CEEA-LR (# C66-136-01).

### Parasite recovery procedure

Golden hamsters (*Mesocricetus auratus*) were infested with 700 cercariae of *Schistosoma mansoni* NMRI strain. Seven weeks post-infection, hamsters’ livers were recovered in saline solution (150 mM NaCl), and ground and eggs were filtered using sieves. Eggs were hatched in sterile fresh water and miracidia were manually collected by pipetting and transferred to a 1.5mL Eppendorf tube for down stream analysis. *Biomphalaria glabrata* (BgBre2 strain) snails were exposed to artificial light (60 W) to enable the snails to shed cercariae which were manually collected by pipetting and transferred to a 1.5mL Eppendorf tube for labeling.

### Optimizing labeling assays

Florescent probes 5-chloromethylfluorescein diacetate (CellTracker™ Green CMFDA, 492/516 nm, C7025, ThermoFisher), 7-amino-4-chloromethylcoumarin (CMAC, DAPI, C2110, 353/466 nm, ThermoFisher) and CellTracker™ red CMTPX (C34552, 577/602nm ThermoFisher) were prepared by dissolving it on DMSO (10 mM stock). On the day of use, a labeling medium was prepared by diluting stock solution to final concentrations: 1µM, 5µM, 10µM, and 100µM. Control labeling was set by exposure to DMSO following the same dye protocol, fixed in 1 to 4% paraformaldehyde for 5 to 10 minutes, and washed twice with phosphate buffered saline (PBS) at room temperature before being placed over the slide and covered with a fluorescence mounting medium (Dako Omnis, GM304). In this study, results and pictures obtained with the green labeling were mainly presented.

### Toxicity assays

Miracidia were exposed for one hour to several concentrations (1µM, 5µM, 10µM, 100µM) of both DMSO-solubilized fluorescent CellTracker™. Control miracidia was either unexposed or exposed to DMSO following the same procedure as that carried out with the different dyes. Non-swimming larvae were counted, viability percentages were calculated and were compared to a control condition (unexposed or DMSO-exposed concentration). Three replicates were performed and at least 20 larvae were counted for each exposure condition.

### Parasites, snails, and compatibility

Compatibility trials between miracidia and *B. glabrata* strains were conducted as previously described (35). For all experimental trials, 20 to 30 snails were infected per condition with 10 miracidia prelabeled with 5µM of fluorescence dye. Two control groups were considered – a negative control, for which snails were infected with unlabeled miracidia in aquarium water and a DMSO control group, for which snails were infected with miracidia exposed to DMSO. Two weeks later, we assessed the prevalence (percentage of infected snails) and intensity (number of developed mother sporocysts per infected snail) of infection for each experimental group. Three independent experiments were performed. Prevalence and intensity were first measured by direct observation of snails after 4 to 5 days of exposure to parasites under fluorescence stereo-microscope (Nikon, SMZ18) or inverted microscope (Nikon, TS100). Then, at 15 days post-exposure, the snail bodies were removed and fixed in modified Raillet-Henry solution to determine the occurrence and the exact number of mother sporocysts established in snail tissue.

### Vibratome section preparation and histolabeling

*B. glabrata* snails are fixed overnight in 4% paraformaldehyde (PAF) after shell removal. Then, fixed snails are washed twice in PBS. Thick sections of snail tissues from foot to digestive glands are cut on the Leica VT1000S vibratome with 0.7 mm/s sectioning speed and 300 to 400-μm thickness in PBS. Thick sections generated by vibratome were observed under fluorescence stereo-microscope and/or confocal microscopy using a Zeiss LSM 700 microscope (Bioenvironment platform from UPVD). For histolabeling analysis, sections are incubated with DAPI (reference 5748, Biotechne) to stain nuclei during 1 min. After 2 washes in PBS, some sections are incubated during 5 to 10 min with Texas Red™-X Phalloidin (591/608 nm, T7471, ThermoFischer) to detect F-actin. After rinsing, tissue sections are mounted on slides with Dako mounting medium to be stored at 4°C. Images obtained with stereo and confocal microscopes are performed with NIS-Elements BR (Nikon) and Zen (Zeiss) softwares, respectively. For confocal microscopy observation, pictures were imported into ImageJ software.

### Statistical analysis

A non-parametric Kruskal-Wallis test (36) was performed followed by a *post-hoc* Dunn test (37) to unveil the potential significant differences occurring between the survival rate of CMAC or CMFDA-exposed and control conditions (unexposed or DMSO-exposed miracidia). A significant difference was considered following a p-value<0.05 for the Kruskal-Wallis test and following p-value<0.025 for the Dunn posthoc test.

## Results

### Free-swimming miracidia toxicity assays

**Figure 1:**
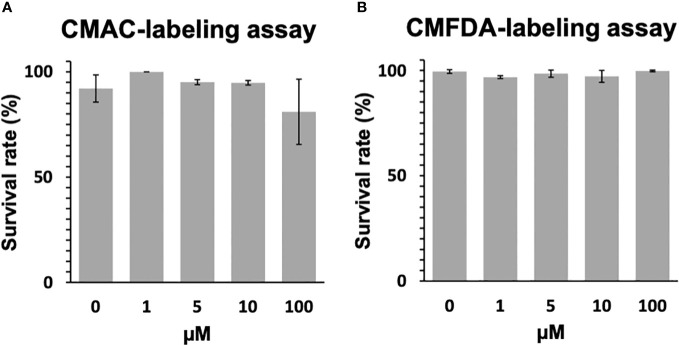
Toxicity assay of CMAC and CMFDA cell trackers. The viability of labeled and unlabeled miracidia populations was assessed in this study. **(A)** The survival rate of CMAC-labeled miracidia slightly decreased when they were exposed to 100 µM for 1 hour. **(B)** The survival rate of CMFDA-labeled miracidia faintly varies between all conditions after 1 hour of exposure. No significant differences were observed between the control and exposed conditions following statistical analysis (p>0.05).

The toxicological effects of CMAC and CMFDA labeling on miracidia of *S. mansoni* were studied by assessing their swimming behavior. For those experiments, the CMAC and CMFDA labeling was performed for 1hr with 1μM, 5μM, 10μM, and 100μM concentrations. For CMAC- and CMFDA-labeled free swimming parasite stages, no significant changes in the survival rate were noticed following both Kruskal-Wallis and Dunn test statistical analysis for miracidia (p>0.05 for all analyzed groups; **Figure 1A, B**).

### Nontoxic fluorescent cell tracker tools are efficient for schistosoma larvae staining

**Figure 2:**
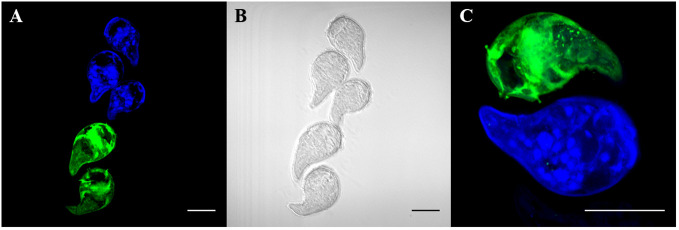
Live labeling of free-swimming miracidia with fluorescent probes CMAC and CMFDA. Fluorescent labeled miracidia were fixed and mounted on slides. Fluorescent labeling was done separately and put together for observation. **(A)** Blue and green fluorescent miracidia after labeling and rinsing. **(B)** Phase contrast microscopy of **(A, C)** High magnification of labeled miracidia. Live miracidia labeled by CMAC (blue) and CMFDA (green). All scale bars indicate 50 µm.

To first determine whether different concentrations of CMAC and CMFDA can be used individually to detect live parasites by fluorescence microscopy, we repeated the same concentrations as in the initial toxicity assay while varied the exposure time from 5 to 60 minutes (data not shown). Furthermore, all live parasites fluoresced when visualized after CMAC or CMFDA uptake with corresponding microscopy imaging of the same miracidia samples providing visual confirmation of a physiological normal phenotype (**Figure 2**, **Video S01**, **S02**, **S03**, **S04**). An optimal concentration of 10µM for 10 minutes was set to obtain maximal efficient dye retention for combining: i) minimum manipulation, ii) sort exposition, and iii) survival rate. These labeling parameters enabled us to archive live labeling without compromising miracidia survival rates for downstream approaches. For example, fluorescent-labeled parasites have the ability to penetrate into snails demonstrating that the labeling time do not seem to reduce their virulence (**Video S05**).

**Figure 3:**
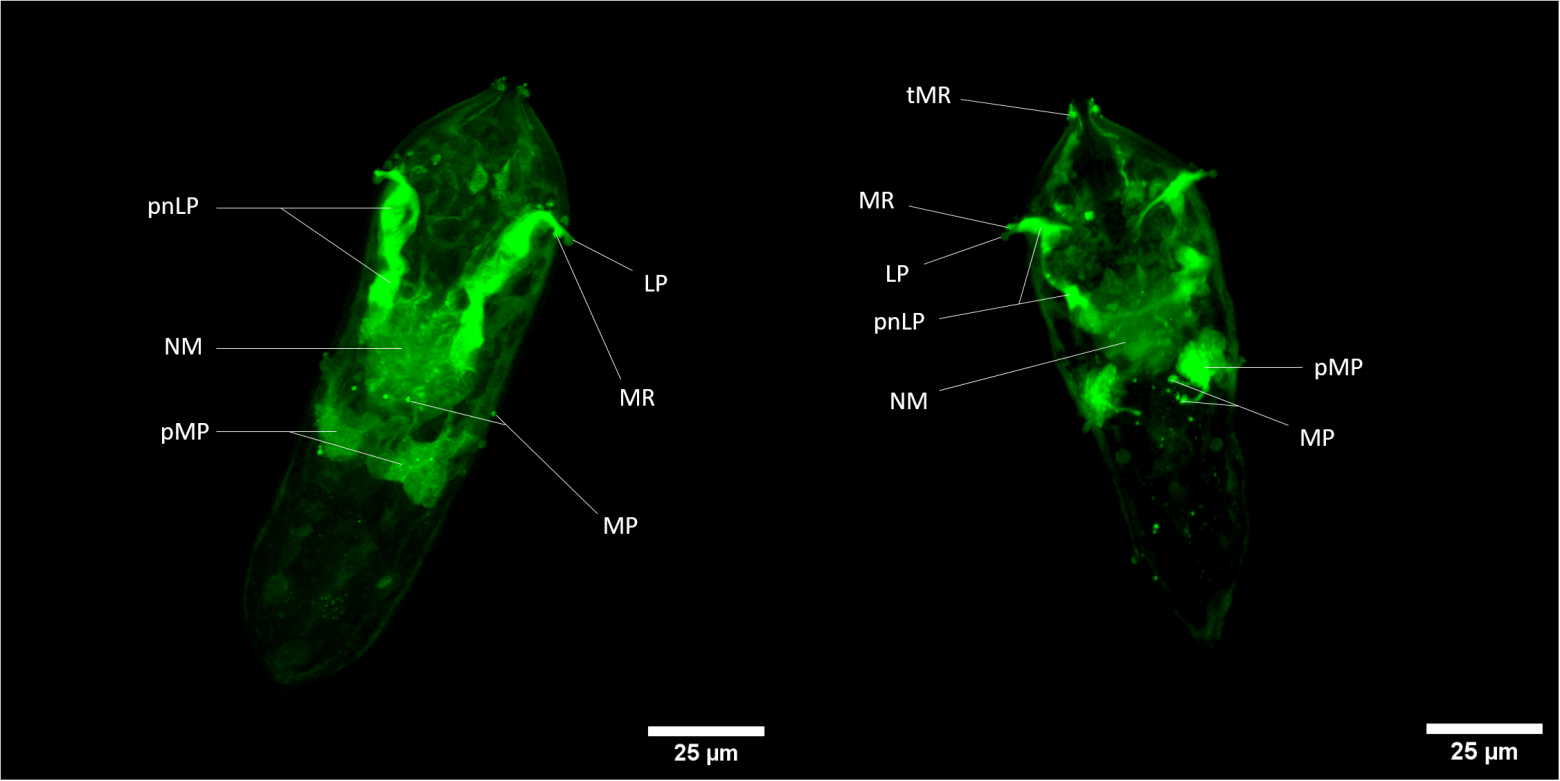
Z-stack of CMFDA-labeled miracidium of *Schistosoma mansoni*. LP, Lateral Papilla; MP, Multiciliated Papilla; MR, Multiciliated Receptor; NM, Neural Mass; pMP, perikaryon of Multiciliated Papilla; pnLP, perikaryon of the neuron to Lateral Papilla; sC, stem Cells; tMR, terebratorial Multiciliated Receptors. Scale bars are 25 µm long.

Briefly, both fluorescent dyes can cross the plasma membrane and remains internalized in some specific cells. For CMFDA probe, it can be metabolized by intracellular esterase into a fluorescent compound that is unable to pass through cell membranes again. Fluorescent labeling is thus sequestered exclusively in cells exhibiting esterase activity. In confocal microscopy, cellular-scale labeling can be explored. Herewith labeled cells related to neural mass can be noticed and with close to greater fluorescence intensity, perikaryon of neurons linked to multiciliated papilla in the median area of the organism (38–40); (**Figure 3**). Also, another perikaryon can be noticeably observed by the 3D reconstitution of a confocal series of pictures with a very strong fluorescence level: neurons related to lateral papillae, and, more precisely, to multiciliated receptors associated with lateral papillae and terebratorial multiciliated receptors (**Video S06**, **S07**) (38, 41).

There is a relatively close correlation of labeled tissues with cells exhibiting acetylcholine esterase activity in miracidia (42) especially neural mass, multiciliated receptors and lateral sides of the terebratorium (strong assumption for multiciliated receptors, that are side orientated) (39, 41). Indeed, this enzyme appeared particularly associated with neural tissues, multiciliated receptors extensions of neuronal cells. The activation of CMFDA could also occur potentially thanks to the presence of acetylcholine esterase in neurons of *S. mansoni* miracidium. This information could also give tools for the study of neural system of this organism and especially its esterase-related activity.

### Fluorescent probes do not affect the miracidia infection and sporocyst development

**Figure 4:**
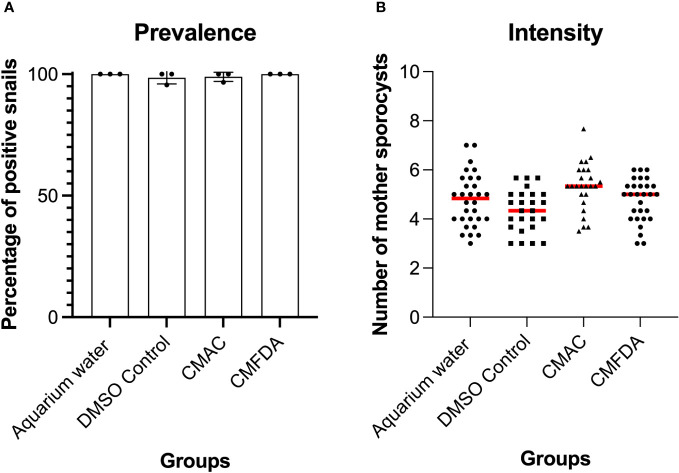
Prevalence **(A)** and intensity **(B)** of Biomphalaria glabrata infected by 10 labeled CMAC, CMFDA, and their respective controls with unlabeled miracidia in aquarium water and a DMSO control group. No significant differences were observed between the control and exposed conditions following statistical analysis (p>0.05).

To further assess the suitability of fluorescent probes for compatibility studies, the prevalence and intensity of live labeled miracidia to *B. glabrata* snails was investigated. *S. mansoni* miracidia were exposed to 10 µM of CMAC and CMFDA or to the respective controls (DMSO or freshwater). Two weeks after infection, the mean prevalence observed in each treated group was higher than 95% (**Figure 4A**). Both labeling probe approaches, along with their respective controls, exhibited no discernible trend in the intensity of infection. Specifically, the intensity of infection, measured as the mean number of sporocysts per infected snail, demonstrated consistency for both the CMAC-labeled group (mean of sporocysts per snail = 5) and the CMFDA-labeled group (mean of sporocysts per snail = 4.6) at a concentration of 10 µM for 10 minutes (**Figure 4B**). Notably, no statistically significant differences emerged in either the prevalence or the intensity of infection across all analyzed groups when compared to their respective controls (p>0.05; **Figures 4A, B**). We performed ANOVA by using the labeling approach as factor, prevalence, and infection intensity as the dependent variables. These findings revealed that the labeling process did not have any statistically significant impact on these variables (p > 0.05). Sporoscyst derived from CMFDA-labeled miracidia showed detectable fluorescence throughout *B. glabrata* snail host infection within the snail body (**Video S08**).

**Figure 5:**
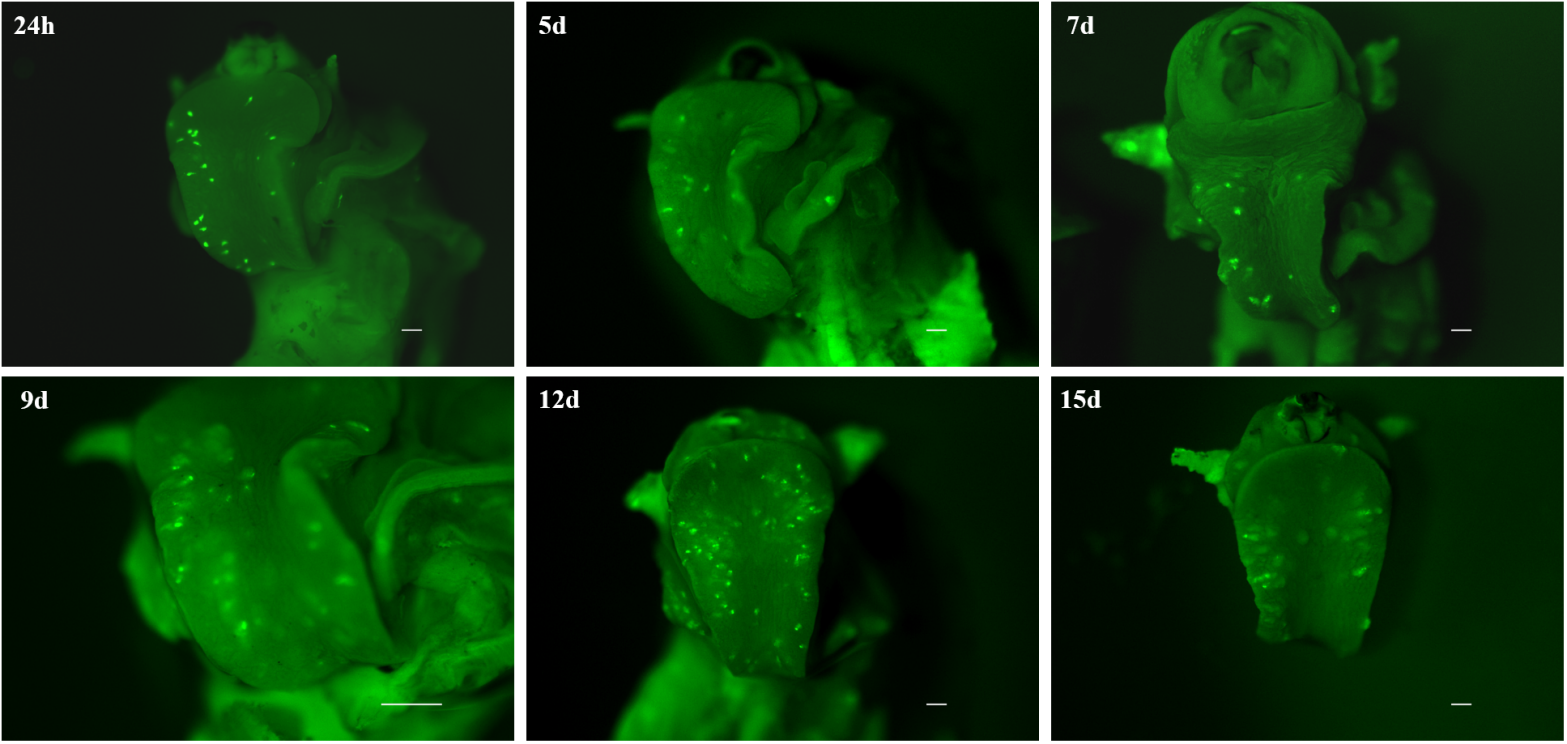
Fluorescence time series monitoring of CMFDA-labeled sporocysts inside Biomphalaria glabrata 24 h, 5 days, 7d ays, 9 days, 12 days, and 15 days post-infection. Parasite location is mainly observed in the head-foot region. All scare bars indicate 500 µm.

By demonstrating that CMFDA-labeled probes could effectively be used to stain live miracidia and newly developed sporocyst, a further experiment was conducted to determine how long CMFDA-labeled miracidia and sporocysts could be detected *in vivo* within *B. glabrata* snail tissue. Here, we demonstrated that fluorescent CMFDA-labeled sporocyst could be easily detected in live snails at least 15 days after infection (**Figure 5**). However, differential staining of individual was supported by observations of fluorescence dilution effect over sporocyst development starting 5 days post-infection (**Figure S1**). At this stage, mother sporocysts, which appeared to developed normally, have a vermiform shape. After 15 days of sporocyst development inside the snails, the fluorescence level of mother sporocyst decreases revealing blisters that seem empty. Consistently, this reduction is associated with the development of daughter sporocysts inside the mother sporocysts. Interestingly, the shape and the fluorescence level in each mother sporocyst differ from one parasite to another showing an asynchronized development and different abilities to differentiate and grow inside the host.

### Other life cycle stages of the parasite are not affected by CMFDA labeling

30 days after infection, direct observations through the translucent shell of living snail demonstrate the presence of daughter sporocysts but unlabeled. Likewise, we no longer observe any fluorescence on the 3-week-old daughter sporocysts collected during their migration to the hepatopancreas and ovotestis area. However, parasites are able to achieve their development cycle. All the parasite-Cell tracker combinations were able to produce cercariae (data not shown, n=16 snails per dye). In addition, these cercariae are also infective and the adult stage are fertile since parasite eggs have been obtained (data not shown, n=3 mice).

### Combination of vital fluorescent dyes and vibratome histological procedure to evaluate *in vivo* host/parasite interactions

**Figure 6:**
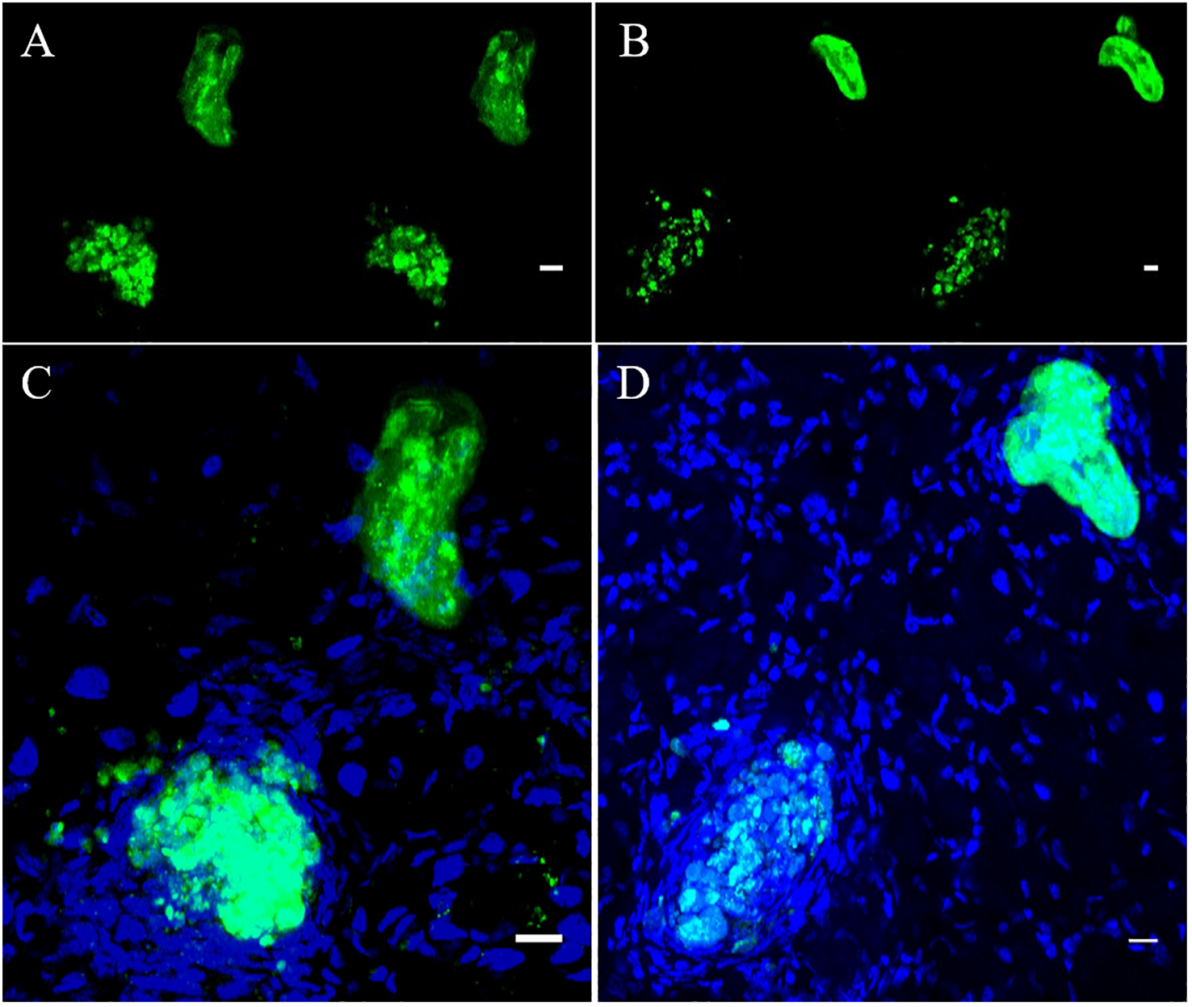
Green fluorescent parasites encapsulated or living inside snail. **(A, B)** are two adjacent focal planes selected from image stack **(C, D)**. which are 2 independent pictures from 2 infected snails obtained by z-stack scanning. Blue stain (DAPI) represents nuclei of snail and parasite cells. All scare bares indicates 10 µm.

**Figure 7:**
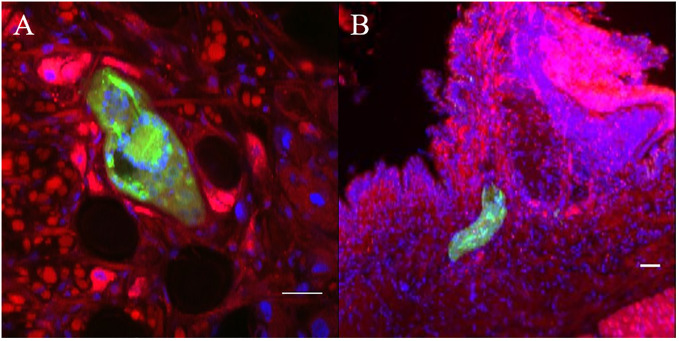
Confocal imaging of Biomphalaria glabrata and Schistosoma mansoni through vibratome sections. **(A, B)** CMFDA-labeled sporocysts were highlighted in green, snail actin filaments were stained with phalloidin in red, and snail cell nuclei were counterstained with DAPI in blue. Scare bares indicates 20 μm.

**Figure 8:**
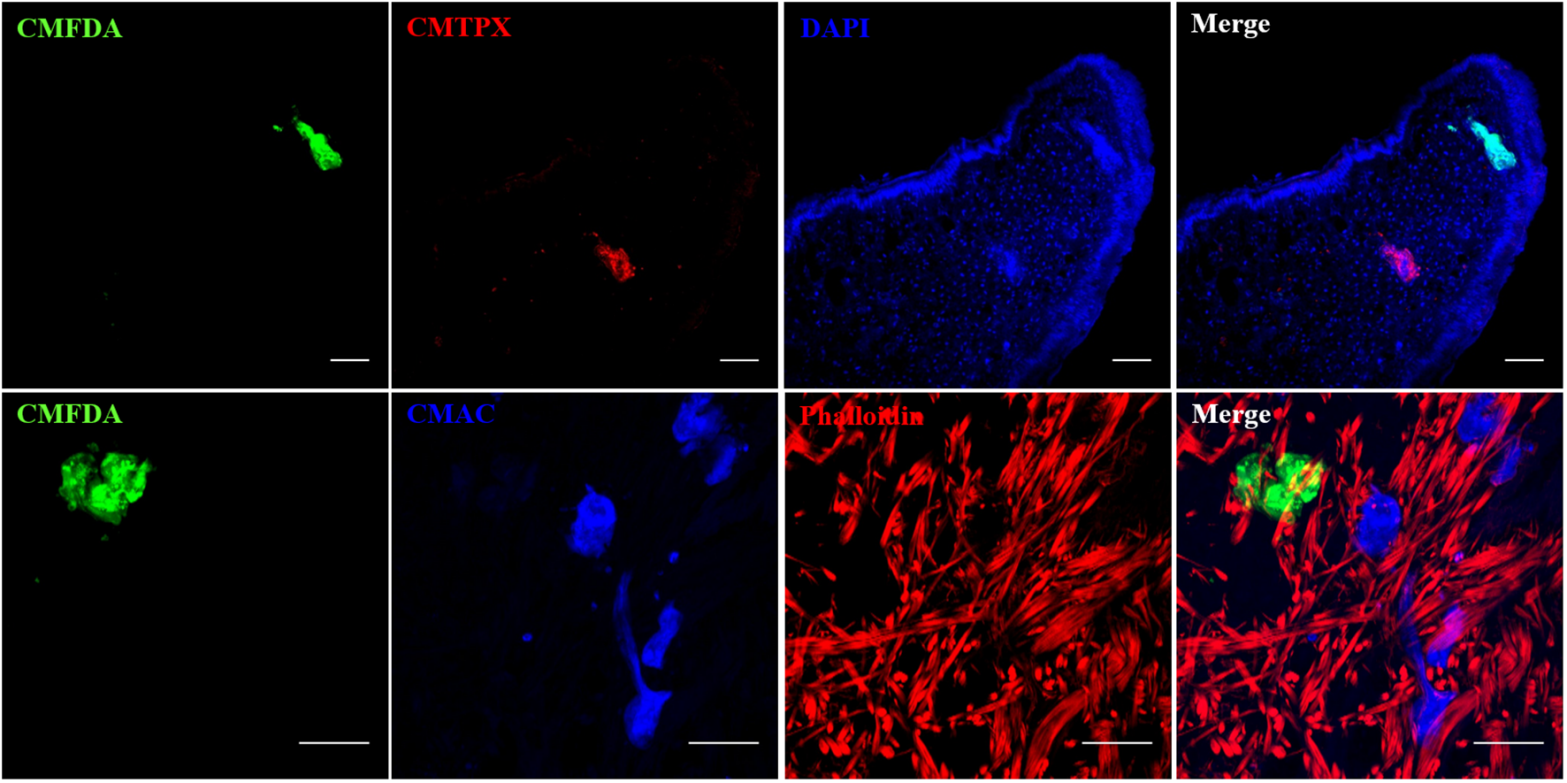
Multiple combinations of parasite labeling. Miracidia were labeled separately with CMFDA, CMTPX or CMAC dye. Then, multi-infestation were performed with differently labeled parasite. Depending on the type of the fluorescent dye, supplementary staining on snail tissue section was carried with DAPI or phalloidin. Scale bar is 50 µm.

Snail/parasite compatibility is characterized by the presence of parasites that are able to develop into mother sporocysts and others that are recognized and killed by the immune system of the host mainly following cellular encapsulation by the hemocytes (snail innate immune cells). Both phenotypes are observed within the same snail exposed to several parasites, some of those miracidia can develop in sporoscyts while others can be immediately recognized and killed by hemocytes or plasmatic factors. No tool exists to separate infected from uninfected BgBAR snails in a exposed population with 20 miradicia of *S. mansoni* venezualian strain for which the prevalence of infection is around 60% (35). The use of dye-labeled parasite make feasible the selection of infected snails within the same population to carry out comparative omic experiments on the first hours of the host/parasite interaction. Infection intensity can vary from 5 to 50% depending on snail-parasite strain combinations used. As vibrating microtome is an effective procedure for generating thick sections that can be used for immunohistochemistry, we employed a robust methodology involving vibratome sectioning and combination with laser scanning confocal microscopy, to observe in 3D the relationships between parasite and snail and thus provide finely comprehensive insights into the studied system. Indeed, after 12h post-infection, encapsulated and damaged parasite forms can be discriminate from developing sporocysts by observing clusters of well-single fluorescent cells (**Figures 6A, B**). The presence of many snail cells probably hemocytes around the parasite reinforces this conclusion (**Figures 6C, D**, **Video S09**, **S10**). Some other immune-cytological labels can also be coupled easily (**Figure 7**, **Video S11**, **S12**). Simple and combined implementation of these different approaches can save time compared to conventional immunohistology techniques requiring paraffin-embedded samples and thin sections (21). Finally, multiple infections with parasites labeled with different fluorescent dyes can be performed in order to study interstrain competition within the same individual snail (**Figure 8**, **Video S13**, **S14**).

## Discussion

The intricate interplay between parasites and their hosts, especially in diseases like schistosomiasis, requires a comprehensive understanding of intermediate host-parasite compatibility that govern their interaction. Understanding the mechanisms that govern the interaction between schistosomes and their intermediate snail hosts is a fundamental requirement for developing efficacious control strategies, as the exponential escalation of parasite numbers heightens the risk of disease propagation. This depth of understanding is essential to develop effective control strategies that can target specific vulnerabilities in the parasite’s life cycle and the host’s immune response. Current methodologies for studying host-parasite compatibility t face strong limitations, hindering a holistic exploration of various strains under diverse environmental conditions. This study introduces a groundbreaking approach that harnesses the potential of fluorescent probes, providing a non-invasive tool for tracking infections and shedding new insights about host-parasite interactions. Besides the notorious rewards of cell trackers for *in-vitro* cell applications, live-labelling have been successfully used as valuable tools for monitoring intracellular parasites for which no genetic tools exist (43). The sensitivity of CMAC and CMFDA are being used in a wide range of context thanks to the property that this fluorescent dye can, i) easily pass through cell membranes, ii) it is well retained in cells, and iii) it passed to daughter cells through several generations but it is not transferred to adjacent cells in a tissue or cell populations.

In this study, we employed fluorescent cell tracker probes to investigate the early stages of sporocyst development within their snail host, by tracking freshwater schistosome larvae stage, the miracidia. Our toxicity assays on free-swimming miracidia, essential for evaluating the viability of our method, underscored the non-toxic nature of fluorescent labeling using CMAC and CMFDA. These probes, whatever the concentrations used, exhibited no significant adverse effects on miracidia survival rates and behavior. This critical finding not only demonstrates the safety of our approach but also lays the foundation for its broad applicability on snail host-parasite studies.

In the past fluorescent probes have yet been used in studies with *S. mansoni* adult worms, but it is the first time that live-labelling was used to evaluate the developmental dynamic from one parasite stage to another as this is the case herein in the transition from freshwater stages, the miracidia to sporocysts in the intermediate snail hosts of *S. mansoni* parasites. Previous studies have used the fluorescent probe resorufin, a substrate for P-glycoprotein (PgP), to evaluate the activity of the excretory system of *S. mansoni* in drug-screening studies (44, 45). Moreover, the fluorescent Hoechst 33258 (bisbenzimide) probe was proposed as a marker of membrane integrity of adult *S. mansoni* worms, due to its affinity for DNA in a context of tegumental lesions (45). Indeed, the evaluation of the presence of tegumental damages and excretory activity in adult worms, were currently under deep investigations in the context of *S. mansoni* resistance to praziquantel, the only available drug to cure schistosomiasis (7, 46–48).

A notable achievement of our method is the extended detectability of fluorescent labeled sporocysts within the snail host. Our results revealed that CMFDA-labeled sporocysts remained detectable for up to 15 days post-infection, significantly advancing the temporal scope of parasite tracking. This temporal resolution is invaluable for unraveling the intricate and evolving nature of schistosoma-snail relationships.

We further deepened our exploration of snail-parasite interactions through vibratome sectioning and histochemistry. This methodology facilitated the visualization of the entire snail body with minimal tissue disruption.

Our research findings have the potential to significantly advance compatibility studies between the snail vector *B. glabrata* and the parasite *S. mansoni*, thereby contributing to the development of novel disease control strategies aimed at reducing the presence of infective parasite forms in freshwater. Furthermore, non-toxic *in vivo* labeling approach introduces an accessible and cost-effective tool that can democratize research in laboratories located in endemic countries. In practical terms, the present work empowers researchers to undertake detailed examinations of first steps of *S. mansoni* development within snail host by employing specific *in vivo* labelling. Besides *B. glabrata-S.mansoni* model compatibility studies, the proposed approach aims to shed light on scenarios that mirror the realities of epidemiologically active sites, where vector snails may encounter, and potentially host, multiple parasite strains and/or species. While studies of coinfection in vector snails are scarce in the literature (49, 50), *in vivo* labelling by fluorescente probes addresses this gap by offering a means to tackle the first steps of parasite infection and development reducing the complexities of studying multiple cycles in laboratory conditions and analyzing the resultant outcomes effectively. In conclusion, this innovative approach holds the potential to unveil valuable insights of compatibility of *B. glabrata-S.mansoni* model and into the intricate dynamics of coinfection, contributing to a deeper understanding of disease transmission and potentially informing more targeted control interventions.

## Data availability statement

The original contributions presented in the study are included in the article/**Supplementary Material**. Further inquiries can be directed to the corresponding authors.

## Author contributions

DD: Conceptualization, Data curation, Formal Analysis, Funding acquisition, Investigation, Methodology, Project administration, Resources, Software, Supervision, Validation, Visualization, Writing – original draft, Writing – review & editing. PP: Conceptualization, Data curation, Formal Analysis, Investigation, Methodology, Validation, Visualization, Writing – review & editing, Software, Writing – original draft. BG: Conceptualization, Data curation, Formal Analysis, Investigation, Methodology, Software, Supervision, Visualization, Writing – review & editing, Writing – original draft. AR: Conceptualization, Investigation, Resources, Writing – review & editing, Data curation, Methodology. RA: Data curation, Investigation, Methodology, Visualization, Writing – original draft, Writing – review & editing, Formal Analysis, Resources, Software, Supervision, Validation.

## Supplementary material

Supplementary Figure 1 High magnification of a green sporocyst 5 days after parasite exposure. The observed sporocyst appears as a misshapen sac characterized by an extremity where fluorescence seems to accumulate. In detail boxes, scare bars are 100 µm.

Supplementary Video 1 Swimming behavior of miracidia after CMFDA labeling and rinsing in Volvic water. Observation was done under stereo-microscope using NIS Element BR software taking an image every 100 msec. Living miracidia can be examined in their natural state without previously being killed by chemical fixation.

Supplementary Video 2 Swimming behavior of miracidia after CMAC labeling and rinsing in Volvic water. Observation was done under stereo-microscope using NIS Element BR software taking an image every 100 msec.

Supplementary Video 3 Miracidia exposure to CMFDA CellTracker in a 1.5ml tube. Observation was done under stereo-microscope using NIS Element BR software.

Supplementary Video 4 Miracidia exposure to CMAC CellTracker in a 1.5ml tube. Observation was done under stereo-microscope using NIS Element BR software.

Supplementary Video 5 Behavior of the CMFDA-labeled live miracidia of *Schistosoma mansoni* penetration *Biomphalaria glabratra* tissue. Note that at the end of the penetration when the parasite is entirely in snail, a lighter region around the parasite is visible suggesting an immediate cellular reaction induced by a changing local environment due to the entry parasite process.

Supplementary Video 6 3-D reconstitution of multiple series confocal pictures from one CMFDA-labeled miracidium. Scale bar: 25µm.

Supplementary Video 7 Unfolding of the different focal plans of a CMFDA-labeled miracidium. Scale bar: 25µm.

Supplementary Video 8 Live green sporocyst of Schistosoma mansoni derived from with CMFDA-labeled miracidia into a living snail Biomphalaria glabrata 3 days after exposition. An image was taken every 0.7 sec.

Supplementary Video 9 Unfolding of the different focal plans of a vibratome cut containing two CMFDA-labeled parasites in host tissues. One is encapsulated (bottom left), the other one is intact (top right). Scale bar: 10 µm.

Supplementary Video 10 Unfolding of the different focal plans of a vibratome cut containing two CMFDA-labeled parasites in host tissues. One is encapsulated (bottom left), the other one is intact (top right). Scale bar: 10 µm.

Supplementary Video 11 3D reconstitution of multiple series confocal pictures from CMFDA-labeled parasite in host tissues. Actin is labeled in red (phalloidin) and nuclei in blue (DAPI). Scale bar: 20µm.

Supplementary Video 12 Unfolding of the different focal plans of a vibratome cut containing one CMFDA-labeled parasite in host tissues. Actin is labelled in red (phalloidin) and nuclei in blue (DAPI). Scale bar: 20 µm.

Supplementary Video 13 Unfolding of the different focal plans of a vibratome cut containing one CMFDA-labeled parasite and one CMTPX-labeled parasite in host tissues. Scale bar: 50µm.

Supplementary Video 14 Unfolding of the different focal plans of a vibratome cut containing one CMFDA-labeled parasite and several CMAC-labeled parasites in host tissues. Actin is labelled in red (phalloidin). Scale bar: 50µm.

## References

[1] Kokaliaris C Garba A Matuska M Bronzan RN Colley DG Dorkenoo AM . Effect of preventive chemotherapy with praziquantel on schistosomiasis among school-aged children in sub-Saharan Africa: a spatiotemporal modelling study. Lancet Infect Dis (2022) 22:136–49. doi: 10.1016/S1473-3099(21)00090-6 PMC8695385 34863336

[2] Vale N Gouveia MJ Rinaldi G Brindley PJ Gärtner F Correia Da Costa JM . Praziquantel for schistosomiasis: single-drug metabolism revisited, mode of action, and resistance. Antimicrob Agents Chemother (2017) 61:e02582–16. doi: 10.1128/AAC.02582-16 PMC5404606 28264841

[3] Shiff C . Why reinvent the wheel? Lessons in schistosomiasis control from the past. PloS Negl Trop Dis (2017) 11:e0005812. doi: 10.1371/journal.pntd.0005812 29073138 PMC5657615

[4] WHO . WHO | Field use of molluscicides in schistosomiasis control programmes: an operational manual for programme managers (2017). WHO. Available at: http://www.who.int/schistosomiasis/resources/9789241511995/en/ (Accessed 3 Jul 2018).

[5] Jourdane J Theron A . Schistosoma mansoni: Cloning by microsurgical transplantation of sporocysts. Exp Parasitol (1980) 50:349–57. doi: 10.1016/0014-4894(80)90038-7 7428911

[6] Wang B Collins JJ Newmark PA . Functional genomic characterization of neoblast-like stem cells in larval Schistosoma mansoni. eLife (2013) 2:e00768. doi: 10.7554/eLife.00768 23908765 PMC3728622

[7] Mouahid G Rognon A de Carvalho Augusto R Driguez P Geyer K Karinshak S . Transplantation of schistosome sporocysts between host snails: A video guide. Wellcome Open Res (2018) 3:3. doi: 10.12688/wellcomeopenres.13488.1 29487916 PMC5806052

[8] Bender RC Goodall CP Blouin MS Bayne CJ . Variation in expression of Biomphalaria glabrata SOD1: A potential controlling factor in susceptibility/resistance to Schistosoma mansoni. Dev Comp Immunol (2007) 31:874–8. doi: 10.1016/j.dci.2006.12.005 17292470

[9] Yoshino TP Dinguirard N Kunert J Hokke CH . Molecular and functional characterization of a tandem-repeat galectin from the freshwater snail Biomphalaria glabrata, intermediate host of the human blood fluke Schistosoma mansoni. Gene (2008) 411:46–58. doi: 10.1016/j.gene.2008.01.003 18280060 PMC2423817

[10] Mourão M Dinguirard N Franco GR Yoshino TP . Role of the Endogenous Antioxidant System in the Protection of Schistosoma mansoni Primary Sporocysts against Exogenous Oxidative Stress. PloS Negl Trop Dis (2009) 3:e550. doi: 10.1371/journal.pntd.0000550 19924224 PMC2771906

[11] Ittiprasert W Miller A Myers J Nene V El-Sayed NM Knight M . Identification of immediate response genes dominantly expressed in juvenile resistant and susceptible Biomphalaria glabrata snails upon exposure to Schistosoma mansoni. Mol Biochem Parasitol (2010) 169:27–39. doi: 10.1016/j.molbiopara.2009.09.009 19815034 PMC2785114

[12] Moné Y Mitta G Duval D Gourbal BEF . Effect of amphotericin B on the infection success of Schistosoma mansoni in Biomphalaria glabrata. Exp Parasitol (2010) 125:70–5. doi: 10.1016/j.exppara.2009.12.024 20067790

[13] Hanington PC Forys MA Loker ES . A somatically diversified defense factor, FREP3, is a determinant of snail resistance to schistosome infection. PloS Negl Trop Dis (2012) 6:e1591. doi: 10.1371/journal.pntd.0001591 22479663 PMC3313920

[14] Portela J Duval D Rognon A Galinier R Boissier J Coustau C . Evidence for specific genotype-dependent immune priming in the lophotrochozoan *biomphalaria glabrata* snail. J Innate Immun (2013) 5:261–76. doi: 10.1159/000345909 PMC6741461 23343530

[15] Pila EA Tarrabain M Kabore AL Hanington PC . A Novel Toll-Like Receptor (TLR) Influences Compatibility between the Gastropod Biomphalaria glabrata, and the Digenean Trematode Schistosoma mansoni. PloS Pathog (2016) 12:e1005513. doi: 10.1371/journal.ppat.1005513 27015424 PMC4807771

[16] Mitta G Gourbal B Grunau C Knight M Bridger JM Théron A . The Compatibility Between Biomphalaria glabrata Snails and Schistosoma mansoni. Adv Parasitol Elsevier (2017) 97:111–45. doi: 10.1016/bs.apar.2016.08.006 28325369

[17] Pila EA Li H Hambrook JR Wu X Hanington PC . Schistosomiasis from a snail’s perspective: advances in snail immunity. Trends Parasitol (2017) 33:845–57. doi: 10.1016/j.pt.2017.07.006 28803793

[18] Li H Hambrook JR Pila EA Gharamah AA Fang J Wu X . Coordination of humoral immune factors dictates compatibility between Schistosoma mansoni and Biomphalaria glabrata. eLife (2020) 9:e51708. doi: 10.7554/eLife.51708 31916937 PMC6970513

[19] Bu L Zhong D Lu L Loker ES Yan G Zhang S-M . Compatibility between snails and schistosomes: insights from new genetic resources, comparative genomics, and genetic mapping. Commun Biol (2022) 5:940. doi: 10.1038/s42003-022-03844-5 36085314 PMC9463173

[20] Galinier R Roger E Moné Y Duval D Portet A Pinaud S . A multistrain approach to studying the mechanisms underlying compatibility in the interaction between Biomphalaria glabrata and Schistosoma mansoni. PloS Negl Trop Dis (2017) 11:e0005398. doi: 10.1371/journal.pntd.0005398 28253264 PMC5349689

[21] Portet A Pinaud S Tetreau G Galinier R Cosseau C Duval D . Integrated multi-omic analyses in Biomphalaria-Schistosoma dialogue reveal the immunobiological significance of FREP-SmPoMuc interaction. Dev Comp Immunol (2017) 75:16–27. doi: 10.1016/j.dci.2017.02.025 28257854

[22] Elhelu O . Susceptibility of snails to infection with schistosomes is influenced by temperature and expression of heat shock proteins. Epidemiol Open Access (2015) 05. doi: 10.4172/2161-1165.1000189 PMC4618387 26504668

[23] Knight M Elhelu O Smith M Haugen B Miller A Raghavan N . Susceptibility of snails to infection with schistosomes is influenced by temperature and expression of heat shock proteins. Epidemiology (Sunnyvale) (2015) 5(2):189. doi: 10.4172/2161-1165.1000189 26504668 PMC4618387

[24] Haggerty CJE Halstead NT Civitello DJ Rohr JR . Reducing disease and producing food: Effects of 13 agrochemicals on snail biomass and human schistosomes. J Appl Ecol (2022) 59:729–41. doi: 10.1111/1365-2664.14087

[25] Lynch AE Noble LR Jones CS Routledge EJ . Common aquatic pollutants modify hemocyte immune responses in Biomphalaria glabrata. Front Immunol (2022) 13. doi: 10.3389/fimmu.2022.839746 PMC9493456 36159819

[26] Paul J Srivastava S Bhattacharya S . Molecular methods for diagnosis of Entamoeba histolytica in a clinical setting: An overview. Exp Parasitol (2007) 116:35–43. doi: 10.1016/j.exppara.2006.11.005 17189632 PMC4247990

[27] Dagley MJ Saunders EC Simpson KJ McConville MJ . High-content assay for measuring intracellular growth of *leishmania* in human macrophages. ASSAY Drug Dev Technol (2015) 13:389–401. doi: 10.1089/adt.2015.652 26247370

[28] Schuster S Krüger T Subota I Thusek S Rotureau B Beilhack A . Developmental adaptations of trypanosome motility to the tsetse fly host environments unravel a multifaceted *in vivo* microswimmer system. eLife (2017) 6:e27656. doi: 10.7554/eLife.27656 28807106 PMC5570225

[29] Kurtz J van der Veen IT Christen M . Fluorescent vital labeling to track cestodes in a copepod intermediate host. Exp Parasitol (2002) 100:36–43. doi: 10.1006/expr.2001.4681 11971652

[30] Trejo-Chávez H García-Vilchis D Reynoso-Ducoing O Ambrosio JR . *In vitro* evaluation of the effects of cysticidal drugs in the Taenia crassiceps cysticerci ORF strain using the fluorescent CellTracker CMFDA. Exp Parasitol (2011) 127:294–9. doi: 10.1016/j.exppara.2010.06.025 20599436

[31] Grego M Stachowitsch M De Troch M Riedel B . CellTracker Green labelling vs. rose bengal staining: CTG wins by points in distinguishing living from dead anoxia-impacted copepods and nematodes. Biogeosciences (2013) 10:4565–75. doi: 10.5194/bg-10-4565-2013

[32] Kilarski WW Martin C Pisano M Bain O Babayan SA Swartz MA . Inherent biomechanical traits enable infective filariae to disseminate through collecting lymphatic vessels. Nat Commun (2019) 10:2895. doi: 10.1038/s41467-019-10675-2 31263185 PMC6603047

[33] Renahan T Sommer RJ . Nematode interactions on beetle hosts indicate a role of mouth-form plasticity in resource competition. Front Ecol Evol (2021) 9:752695. doi: 10.3389/fevo.2021.752695

[34] Ittiprasert W Moescheid MF Chaparro C Mann VH Quack T Rodpai R . Targeted insertion and reporter transgene activity at a gene safe harbor of the human blood fluke, Schistosoma mansoni. Cell Rep Methods (2023) 3:100535. doi: 10.1016/j.crmeth.2023.100535 37533651 PMC10391569

[35] Theron A Rognon A Gourbal B Mitta G . Multi-parasite host susceptibility and multi-host parasite infectivity: a new approach of the Biomphalaria glabrata/Schistosoma mansoni compatibility polymorphism. Infect Genet Evol J Mol Epidemiol Evol Genet Infect Dis (2014) 26:80–8. doi: 10.1016/j.meegid.2014.04.025 24837670

[36] Kruskal WH Wallis WA . Use of ranks in one-criterion variance analysis. J Am Stat Assoc (1952) 47:583–621. doi: 10.1080/01621459.1952.10483441

[37] Dunn OJ . Multiple comparisons using rank sums. Technometrics (1964) 6:241–52. doi: 10.1080/00401706.1964.10490181

[38] Pan SC . The fine structure of the miracidium of Schistosoma mansoni. J Invertebr Pathol (1980) 36:307–72. doi: 10.1016/0022-2011(80)90040-3 7452064

[39] Eklu-Natey DT Wüest J Swiderski Z Striebel HP Huggel H . Comparative scanning electron microscope (SEM) study of miracidia of four human schistosome species. Int J Parasitol (1985) 15:33–42. doi: 10.1016/0020-7519(85)90098-0 3980140

[40] Samuelson JC Caulfield JP . Role of pleated septate junctions in the epithelium of miracidia of Schistosoma mansoni during transformation to sporocysts *in vitro* . Tissue Cell (1985) 17:667–82. doi: 10.1016/0040-8166(85)90003-5 4060143

[41] Poteaux P Gourbal B Duval D . Time series analysis of tegument ultrastructure of *in vitro* transformed miracidium to mother sporocyst of the human parasite Schistosoma mansoni. Acta Trop (2023) 240:106840. doi: 10.1016/j.actatropica.2023.106840 36681315

[42] Bruckner DA Voge M . The nervous system of larval schistosoma mansoni as revealed by acetylcholinesterase staining. J Parasitol (1974) 60:437–46. doi: 10.2307/3278359 4833856

[43] Boleti H Ojcius DM Dautry-Varsat A . Fluorescent labelling of intracellular bacteria in living host cells. J Microbiol Methods (2000) 40:265–74. doi: 10.1016/S0167-7012(00)00132-9 10802143

[44] Sato H Kusel JR Thornhill J . Functional visualization of the excretory system of adult Schistosoma mansoni by the fluorescent marker resorufin. Parasitology (2002) 125:527–35. doi: 10.1017/s0031182002002536 12553571

[45] Couto FFB Coelho PMZ Araújo N Kusel JR Katz N Mattos ACA . Use of fluorescent probes as a useful tool to identify resistant *Schistosoma mansoni* isolates to praziquantel. Parasitology (2010) 137:1791–7. doi: 10.1017/S003118201000065X 20561394

[46] Liang Y-S Wang W Dai J-R Li H-J Tao Y-H Zhang J-F . Susceptibility to praziquantel of male and female cercariae of praziquantel-resistant and susceptible isolates of Schistosoma mansoni. J Helminthol (2010) 84:202–7. doi: 10.1017/S0022149X0999054X 19765323

[47] Buchter V Hess J Gasser G Keiser J . Assessment of tegumental damage to Schistosoma mansoni and S. haematobium after *in vitro* exposure to ferrocenyl, ruthenocenyl and benzyl derivatives of oxamniquine using scanning electron microscopy. Parasit Vectors (2018) 11:580. doi: 10.1186/s13071-018-3132-x 30400935 PMC6219169

[48] Wendt GR Collins JN Pei J Pearson MS Bennett HM Loukas A . Flatworm-specific transcriptional regulators promote the specification of tegumental progenitors in Schistosoma mansoni. eLife (2018) 7:e33221. doi: 10.7554/eLife.33221 29557781 PMC5927768

[49] Bonfim TCDS Tunholi-Alves VM Martins FG Mota EM Maldonado A Pinheiro J . Biological and histological changes in reproductive patterns of Biomphalaria glabrata experimentally co-infected by Echinostoma paraensei and Angiostrongylus cantonensis. Exp Parasitol (2018) 195:66–70. doi: 10.1016/j.exppara.2018.10.005 30401655

[50] Bonfim TCDS Martins FG Tunholi-Alves VM Lima MG Mota EM Maldonado A . Evaluation of changes in the carbohydrate metabolism of Biomphalaria glabrata Say 1818 exposed to experimental coinfection by Angiostrongylus cantonensis (Nematoda) and Echinostoma paraensei (Trematoda). J Invertebr Pathol (2020) 170:107314. doi: 10.1016/j.jip.2019.107314 31866115

