# Supplementary figures and images for: Fluorescent non transgenic schistosoma to decipher host-parasite phenotype compatibility
Source: Front Immunol. 2023 Nov 27;14:1293009. doi: 10.3389/fimmu.2023.1293009 (PMC10721968; doi:10.3389/fimmu.2023.1293009)

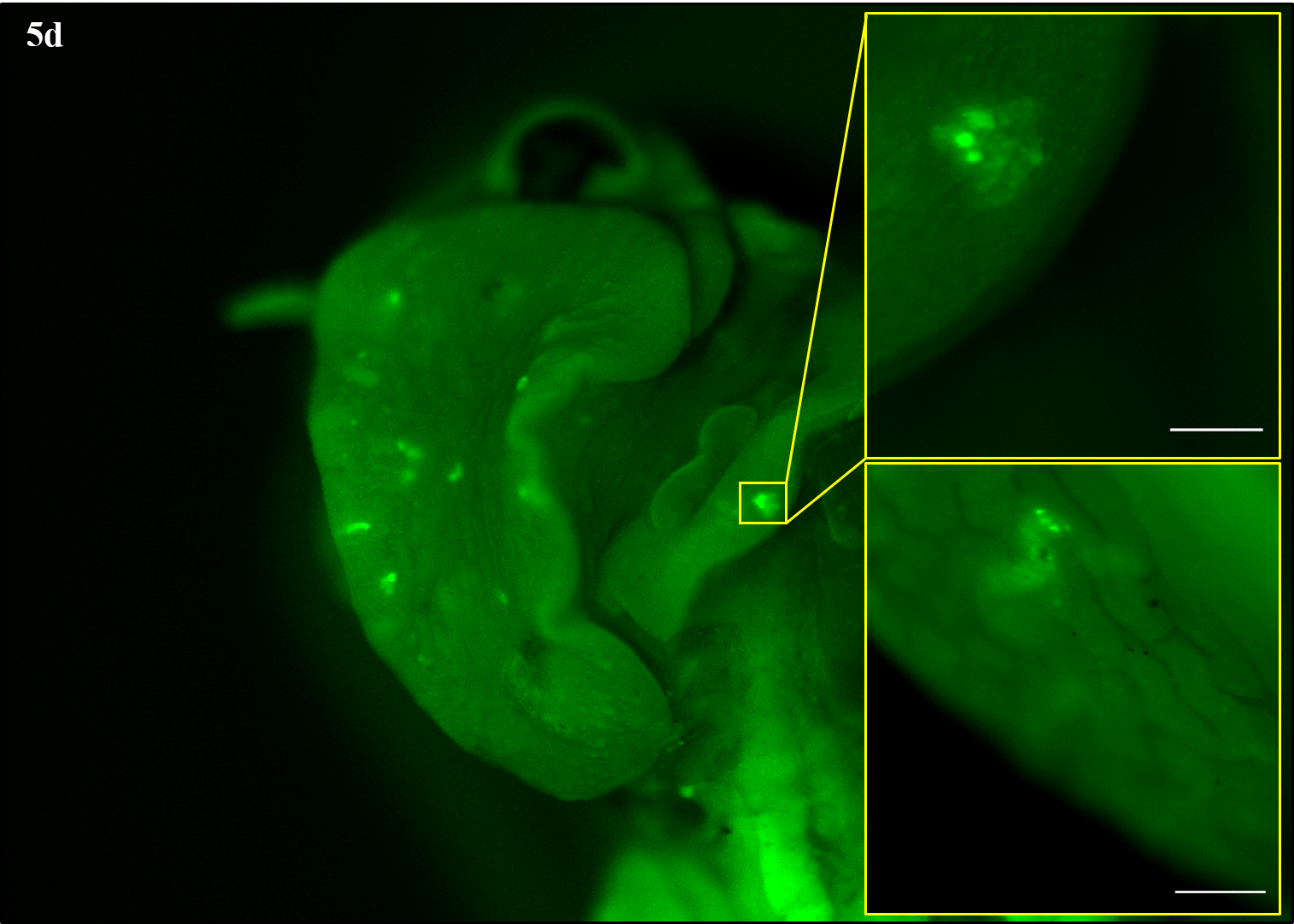

Supplement: Supplementary Figure 1 — High magnification of a green sporocyst 5 days after parasite exposure. The observed sporocyst appears as a misshapen sac characterized by an extremity where fluorescence seems to accumulate. In detail boxes, scare bars are 100 µm. [file Image_1.tif]
